# Structural studies of the nucleus-like assembly of jumbo bacteriophage 201φ2-1

**DOI:** 10.3389/fmicb.2023.1170112

**Published:** 2023-04-17

**Authors:** Zhe Liu, Ye Xiang

**Affiliations:** Center for Infectious Disease Research, Beijing Frontier Research Center for Biological Structure & Beijing Advanced Innovation Center for Structural Biology, Department of Basic Medical Sciences, School of Medicine, Tsinghua University, Beijing, China

**Keywords:** jumbo bacteriophage, nucleus-like shell, dynamic assembly, lattice-like structure, cube-like assembly, tetramer

## Abstract

The jumbo phages encode proteins that assemble to form a nucleus-like compartment in infected cells. Here we report the cryo-EM structure and biochemistry characterization of gp105, a protein that is encoded by the jumbo phage 201φ2-1 and is involved in the formation of the nucleus-like compartment in phage 201φ2-1 infected *Pseudomonas chlororaphis*. We found that, although most gp105 molecules are in the monomeric state in solution, a small portion of gp105 assemble to form large sheet-like assemblies and small cube-like particles. Reconstruction of the cube-like particles showed that the particle consists of six flat head-to-tail tetramers arranged into an octahedral cube. The four molecules at the contact interface of two head-to-tail tetramers are 2-fold symmetry-related and constitute a concave tetramer. Further reconstructions without applying symmetry showed that molecules in the particles around the distal ends of a 3-fold axis are highly dynamic and have the tendency to open up the assembly. Local classifications and refinements of the concave tetramers in the cube-like particle resulted in a map of the concave tetramer at a resolution of 4.09 Å. Structural analysis of the concave tetramer indicates that the N and C terminal fragments of gp105 are important for mediating the intermolecular interactions, which was further confirmed by mutagenesis studies. Biochemistry assays showed that, in solution, the cube-like particles of gp105 are liable to either disassemble to form the monomers or recruit more molecules to form the high molecular weight lattice-like assembly. We also found that monomeric gp105s can self-assemble to form large sheet-like assemblies *in vitro*, and the assembly of gp105 *in vitro* is a reversible dynamic process and temperature-dependent. Taken together, our results revealed the dynamic assembly of gp105, which helps to understand the development and function of the nucleus-like compartment assembled by phage-encoded proteins.

## Introduction

The jumbo bacteriophages have a large dsDNA genome of 200–500 kbp that encodes 64–675 proteins, including enzymes and proteins required for their genome replication and assembly of the virion (Yuan and Gao, 2017). Some jumbo bacteriophages produce nucleus-like compartments in host cells, which provide a special location for the phage genome replication, packaging and protect phage genomes from host cell enzyme degradation (Chaikeeratisak et al., 2017a,b, 2021; Guan and Bondy-Denomy, 2020). The nucleus-like compartments are assembled mainly by a phage-encoded nucleus-like shell protein (NLSP). At the early time of infection, NLSP is produced in a large amount and is uniformly distributed in infected cells (Chaikeeratisak et al., 2017b). Then, triggered by an unknown factor, NLSPs assemble to form a nucleus-like structure at the polar of the infected cell, where the nucleus-like shell encapsulates the released phage genome (Chaikeeratisak et al., 2017b,^2019^). The nucleus-like shell is then translocated to the center of the infected cell with the help of tubulin-like structures assembled by a phage-encoded tubulin protein (phuZ) (Kraemer et al., 2012).

Enzymes relevant to phage genome replication and phage gene transcription were identified in the nucleus-like shell, whereas those relevant to the translation of phage proteins occur exclusively outside the nucleus-like shell (Chaikeeratisak et al., 2017b). Thus, similar to the nucleus compartments in eukaryotic cells, the phage nucleus-like shell is proposed to function in separating the phage genome replication and gene transcription from the phage protein translation. The nucleus-like shells were shown to selectively import certain phage proteins (Nguyen et al., 2021). The assembled procapsids were found to anchor around the nucleus-like shell for genome packaging (Chaikeeratisak et al., 2017b,^2019^). Mechanisms involved in the assembly of the phage nucleus-like shell and the selective encapsidation of materials in the phage nucleus-like shell are yet to be determined.

Bacteriophage 201φ2-1 is a jumbo phage that has a genome of 316 kbp and infects the bacterial species *Pseudomonas chlororaphis* (Thomas et al., 2008). Previous studies using mass spectrometry analysis identified the phage-encoded gene product 105 (gp105) as the major component of the nucleus-like shell (Chaikeeratisak et al., 2017b). Gp105 has 631 amino acids and shares sequence homology with other NLS proteins (Chaikeeratisak et al., 2017a,b). A cryo-EM structure of gp105 was reported recently (Laughlin et al., 2022). Here we report the cryo-EM structural and biochemistry studies of the NLS protein gp105 from phage 201φ2-1. Our results revealed the dynamic structure of gp105 and the results can help to explain the dynamic assembly of the nucleus-like shell. Furthermore, our biochemistry data showed that temperature is a key factor that affects the assembly of gp105.

## Results

### Production and characterization of the nucleus-like shell protein (NLSP) gp105

We produced in *Escherichia coli* a His-tagged recombinant gp105 of phage 201φ2-1, which has a His6 tag fused at the C terminus. Size-exclusion chromatography (SEC) analysis of the affinity purified gp105 showed three peaks (Supplementary Figures 1A, B). The different elution positions of the peaks indicate three different oligomeric states of gp105, including the highly aggregated gp105 eluted at the void volume, gp105 in an oligomeric state with a molecular weight of approximately 1.97 MDa, and gp105 in a monomeric state (Supplementary Figure 1C). The peak of the monomeric gp105 is significantly higher than the peaks of the other two forms. The eluted peaks from SEC were then subjected to cryo-electron microscopy (cryo-EM) analysis.

**FIGURE 1:**
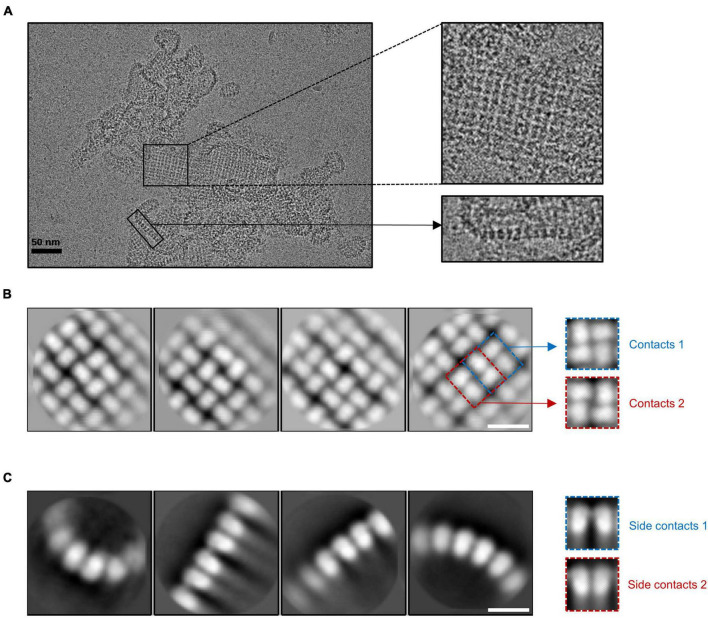
The lattice-like structure of gp105. **(A)** A representative cryo-EM micrograph of the highly aggregated gp105 eluted at the void volume of the size exclusion chromatography (SEC). The insets showing the zoom-in pictures of the central part and edge part, respectively. **(B,C)** 2D class averages of the highly aggregated gp105 eluted at the void volume of SEC. The averaged images of the particles located in the aggregates showing the top view of the assembly **(B)**. The averaged images of the particles located at the edge of the aggregates showing the side view of the assembly **(C)**. The scale bars represent 10 nm.

The cryo-EM results showed that gp105 eluted at the void volume forms large sheets, in which gp105 molecules are arranged into lattice-like arrays (Figure 1A). We selected different subregions from the sheets and then performed several rounds of reference-free 2D classifications (Supplementary Figure 2). The images in the same class were averaged to improve the signal to noise ratio. Some of the 2D class averaged images showed clear lattices of P4 symmetry, in which two different contacting environments could be identified (Figure 1B). In one of the contacting environments, monomers in the tetramer are 4-fold symmetry related and have head-to-tail interactions. The tetramer with head-to-tail interactions is the repeating unit of the sheet with the P4 symmetry (Figure 1B, contacts 1), while in the other contacting environment, monomers in the tetramer are 2-fold symmetry related and interact with each other through one end (Figure 1B, contacts 2). Sizes of the gaps between the tetramers vary among images from different classes, which may be a reflection of the different curvatures of the sheets. The head-to-tail tetramers have a size of approximately 100 Å × 100 Å, and each molecule in the tetramer has a size of approximately 60 Å × 40 Å (Figure 1B). At the edge of the sheets, the molecules have a different arrangement (Figure 1A). Two-dimensional image analysis near the edge of the sheets showed likely the tilted side view of the assemblies, in which the molecules are aligned in lines of different curvatures and the length of the molecule is approximately 70 Å (Figure 1C). The arrangement of the molecules in side views at the edge indicates that the sheets are not open sheets but most likely collapsed closed shells. Taken together, a gp105 monomer has a shape roughly resembling a cuboid with a size of approximately 60 Å × 40 Å × 70 Å. From the side views, two different types of interactions between the molecules could be identified, in which one molecule interacts with one adjacent molecule through its top part, while interacting with the other adjacent molecule through its bottom tail part (Figure 1C). The averaged images of the side views also showed that the assembled molecules present different curvatures, which could lead to the assembly of a closed shell (Figure 1C).

### Structure of the gp105 cube-like assembly

**FIGURE 2:**
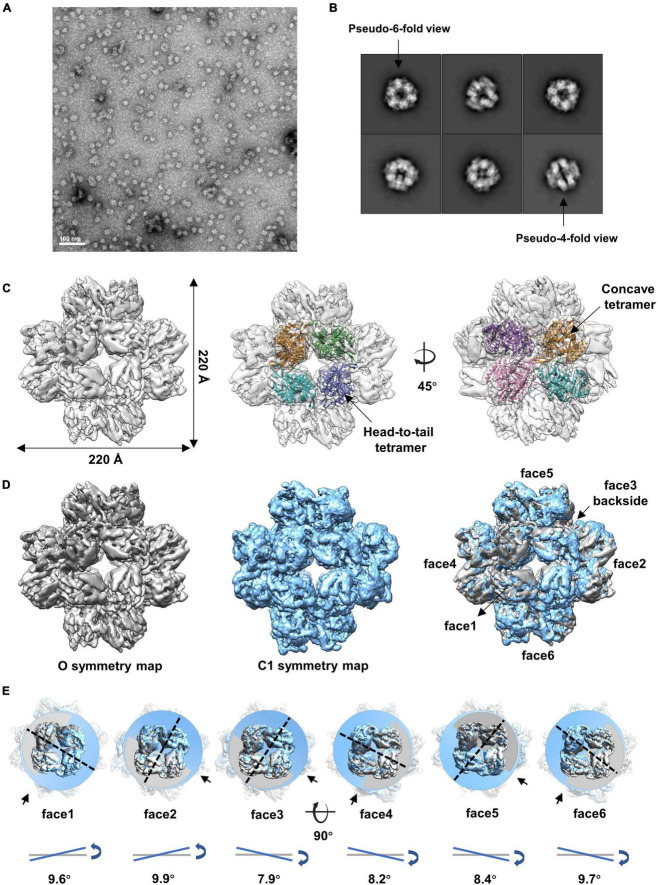
Cryo-EM reconstructions of the small cube-like gp105 particles calculated with O and C1 symmetry, respectively. **(A)** A representative negative-staining micrograph showing the particles of gp105 from the peak2 of SEC. **(B)** 2D class averages of the gp105 cube-like assembly. **(C)** Surface rendered representations showing the cryo-EM map of the gp105 cube-like assembly reconstructed with O symmetry imposed (O map). The fitted gp105 (residues 61–577) structures show the head-to-tail (middle) and concave tetramers (right). The gp105 structure was predicted by using AlphaFold and each gp105 monomer is colored differently. The map is contoured at 2.3 σ. (**D**, Left) Surface rendered representations showing the cryo-EM map of the gp105 cube-like assembly reconstructed with O symmetry imposed (O map). (Middle) Surface rendered representation showing the cryo-EM map of the gp105 cube-like assembly reconstructed without applying any symmetry (C1 map). (Right) Superimposition of the C1 and O maps. The maps were superimposed by maximizing the map correlation coefficient of the aligned maps with the Fit in Map function of Chimera. Different faces of the octahedron are marked by different numbers. The C1 map is contoured at 2.3 σ and the O map is contoured at 2.4 σ. **(E)** Analysis of the superimposed maps at each face showing the positional deviation of the head-to-tail tetramers in the C1 map relative to these in the O map. The planes were generated with the fitted models of the head-to-tail tetramers and the Structure Measurements function of Chimera. The tilt angles are calculated with the planes.

We then analyzed the peak eluted at 11.4 ml from the SEC (Supplementary Figure 1A). The results showed that gp105 molecules in the 11.4 ml peak form mainly small particles with a diameter of approximately 22 nm (Figure 2A). Two-dimensional image analysis of the selected particles showed two major views of the particles, including the pseudo-4-fold views and the pseudo-6-fold views (Figure 2B). The observed symmetric views are an indication of dihedral, octahedral, or tetrahedral symmetry of the particles. We used the two symmetric views as the top and side views of the particle to generate an initial model with RELION (Scheres, 2012), and then use the initial model as a reference model for 3D classifications with either dihedral, octahedral, or tetrahedral symmetry imposed. Only the octahedral (O) symmetry gave a reasonable result. Thus, we calculated the reconstruction with O symmetry imposed and the resolution of the reconstruction measured was 4.76 Å (Supplementary Figures 3A, B). Overall, the particle has a cube-like structure with a size of 220 Å × 220 Å × 220 Å and is composed of 24 gp105 molecules (Figure 2C). The 24 gp105 molecules assemble to form 6 flat head-to-tail tetramers that constitute the 6 faces of the particle. Features of the flat head-to-tail face tetramers are consistent with the head-to-tail tetrameric subunit in the sheets (Figure 2C). Molecules in the head-to-tail face tetramer are oriented roughly perpendicular to each other. The four molecules at the interface of two head-to-tail tetramers sit on a 2-fold axis, interact with each other through one distal end of the molecule and form a concave tetramer (Figure 2C).

**FIGURE 3:**
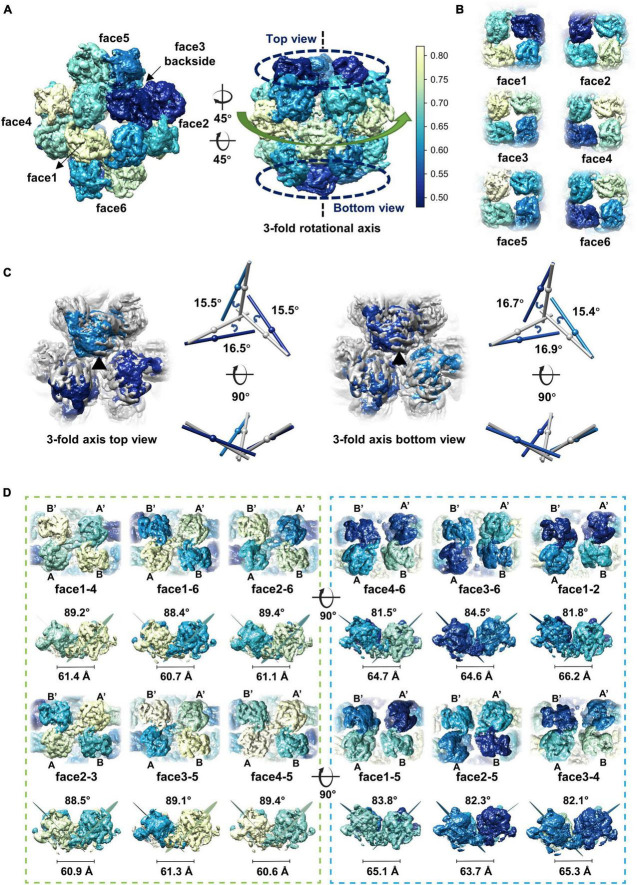
Dynamics of the gp105 monomers in the cube-like assembly. **(A)** Surface rendered representations showing the cryo-EM maps with the C1 symmetry oriented around the 4-fold rotational axis (left) and the 3-fold rotational axis (right), respectively. Each molecule is colored according to the map CC values calculated with the superimposed C1 and O maps. The C1 map presented is contoured at 2.3 σ. **(B)** Surface rendered representations showing the six head-to-tail tetramers. Each molecule is colored according to the map CC values calculated with the superimposed C1 and O maps. (**C**, Left) Surface rendered representations showing the top and bottom of the superimposed assemblies oriented around the 3-fold axis. Molecules in the O map are colored gray. Molecules in the C1 map are colored according to the CC values from light blue to dark blue. (Right) Comparisons of the long axes of the molecules in the O and C1 maps. The long axes of the molecules in the O and C1 maps are colored gray and blue, respectively. **(D)** Surface rendered representations showing all the concave tetramers in the map with C1 symmetry. The bending angle between the planes and the distance between the gravity centers of the two symmetrical subunits AB’ and A’B on the concave tetramers were calculated and shown.

Although different classification strategies were used, high resolution structure of the assembly could not be obtained with mandatory octahedral symmetry imposed. One possible reason is that the molecules are not strictly following the symmetry imposed. Therefore, we further calculated the reconstruction without applying any symmetry to a resolution of 6.41 Å (Supplementary Figures 3A, B). By superimposing the map without applying any symmetry into the map with the O symmetry imposed, we found that the head-to-tail tetramers in the map without applying any symmetry have an average tilt angle of approximately 9 degrees when compared to these that have been averaged with the octahedral symmetry (Figures 2D, E). To find out the intensity of variation for each molecule, we calculated the correlation coefficients of molecules at each symmetry-related equivalent position separately. The results showed that, when oriented around a 3-fold axis, molecules at the top and bottom of the assembly deviate dramatically from the octahedral symmetry-related positions as indicated by the poor CC values (Figures 3A, B), while molecules in the middle are more stable and are maintained by interactions within the concave tetramers (Figure 3A). Molecules at the top and bottom tend to tilt outward and open up the assembly compared with the map with the O symmetry imposed (Figures 3A, C). The largest tilt angle of a single monomer is approximately 17 degrees (Figure 3C). Molecules in the middle of the assembly belong to 12 concave tetramers and can be divided into two groups (Figure 3D). In one group, molecules in the tetramer have similar and minor deviations from the ideal positions, and the 2-fold symmetry among the molecules is still maintained. In the other group, molecules in the tetramer have significantly different and large deviations from the ideal positions, and the 2-fold symmetry among the molecules has been disrupted (Figure 3D). The bending angle between the planes calculated with the subunits AB’ and A’B of the concave tetramers, respectively, vary from 89 degrees to 81 degrees (Figure 3D). The distance between the gravity centers of subunits AB’ and A’B increases as the bending angle decreases (Figure 3D). Concave tetramers with larger deviations and smaller bending angles lack the intermolecular interactions as indicated by the missing densities at the interface (Figure 3D). Decrease in the bending angles between the molecules would reduce the curvatures and lead the molecules to form the flat lattice-like assemblies.

### Structure of the gp105 concave tetramer

**FIGURE 4:**
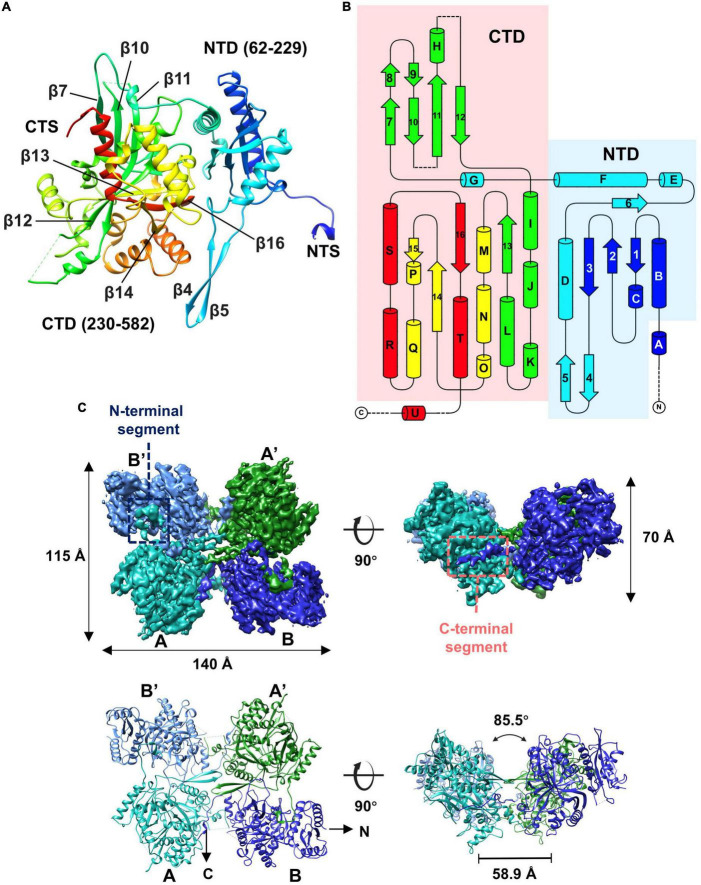
Overall structure of the gp105 concave tetramer. **(A)** Ribbon diagrams showing the structure of a gp105 monomer (molecule A) in the concave tetramer. The structure is colored as a rainbow from the N terminus (blue) to the C terminus (red). The N-terminal domain is colored from blue to cyan and the C-terminal domain is colored from green to red. The dashed lines represent unresolved loops. **(B)** Diagrams showing the topology of gp105. **(C)** Surface rendered representations and ribbon diagrams showing the overall structure of the concave tetramer. Left-top is the top view of the gp105 concave tetramer assembly, and the right-top is the front view of the gp105 concave tetramer. The structure is shown as a ribbon diagram with the N terminus and C terminus labeled. The map is contoured at 4.3 σ. Molecule A is colored cyan, molecule B is colored dark blue, molecule A’ is colored green, and molecule B’ is colored light blue. AB’ and A’B are the symmetry-related subunits.

Given that some of the concave tetramers can have a stable conformation, we performed locally focused classifications and refinements on the concave tetramers with C2 symmetry imposed. Through several rounds of classifications and refinements, we finally obtained a reconstruction of the concave tetramer at a resolution of 4.09 Å (Supplementary Figures 3, 4). The density map is good enough for us to build a model with the structure predicted by AlphaFold as a reference (Jumper et al., 2021). The structure of gp105 is composed of N- and C- two domains (Figures 4A, B). The N-terminal domain (NTD) contains a β sheet core of 4 antiparallel β strands that is sandwiched by 3 α helices. The C-terminal domain (CTD) contains a twisted β sheet core of 7 antiparallel β strands that is sandwiched by 10 α helices (Figures 4A, B).

**FIGURE 5:**
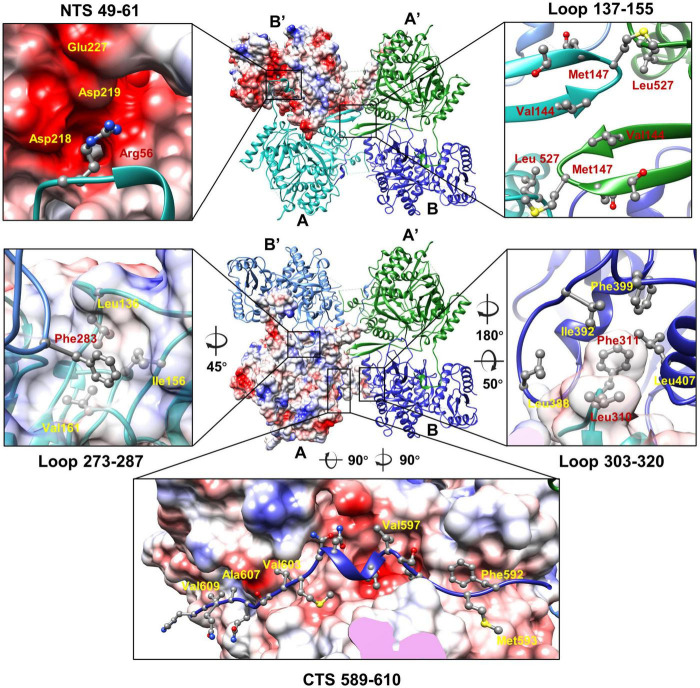
The interactions between the monomers of the gp105 concave tetramer. Top: The molecule B’ is shown in a surface rendered representation. Other three molecules are shown in ribbon and colored cyan (molecule A), green (molecule A’), and dark blue (molecule B). Bottom: The molecule A is shown in a surface rendered representation. Other three monomers are shown in ribbons and colored green (molecule A’), dark blue (molecule B), and light blue (molecule B’). The surface is colored according to the surface electrostatic potential with red representing negative electrostatic potential and blue representing positive electrostatic potential. The key residues at the interface are shown in balls and sticks.

Structures of the two unique molecules in the concave tetramer have differences mainly in loops 273–287, 303–320, 137–155, the N-terminal fragment 49–63, and the C-terminal fragment 582–610 (Figure 4C and Supplementary Figure 5A). Loop 137–155 is located in between helix D and β 3 and contains a β hairpin structure protruding from the NTD. The hairpin has direct interactions with that from the symmetry-related molecule and is rich in hydrophobic residues, among which residues V144, M147, and L527 form a hydrophobic core with these from the symmetry-related molecule (Figure 5). Interactions between molecule A and the two neighboring molecules B and B’ are mediated mainly by loop 303–320 (molecule A), loop 273–287 (molecule B’), the N-terminal segment (residues 49–61, molecule A), and the C-terminal fragment (residues 589–610, molecule B) (Figure 5). The loop 303–320 of molecule A contains a short α helix that has two turns. The distal end of the short α helix has three hydrophobic residues L310, F311, and P312, which are in close contact with a hydrophobic surface groove constituted by residues L388, I392, F399, L407 of the neighboring molecule B (Figure 5). Loop 273–287 from the neighboring molecule B’ is inserted into a hydrophobic surface pocket of molecule A. Residue F283 of loop 273–287 is located at the center of the surface hydrophobic pocket that is constituted by residues L136, I156, and V161 of molecule A. The N-terminal segment (NTS: residues 49–61) of molecule A is inserted into a groove between the NTD and CTD of the neighboring molecule B’. The groove is rich in negatively charged residues and is mainly constituted by residues D218, D219, and E227 of molecule B’. The side chain of R56 of the N-terminal segment 49–61 is in close contact with the two aspartic acid residues in the pocket (Figure 5). The N-terminal segment mediated interactions are the same as these in between the monomers of the head-to-tail tetramer. The C-terminal segment (CTS: residues 589–610) of molecule B is inserted into a groove of the CTD from molecule A. Hydrophobic residues, including F592, M593, V597, V603, A607, and V609, are involved in the interactions between molecule A and the CTS of molecule B. The interaction mediated by the CTS, the loop 303–320, and loop 137–155 are unique and only exist in between the molecules of the concave tetramers (Figure 5).

In the high-resolution concave tetramer structure we obtained, the angle between the planes calculated with the subunits AB’ and A’B, respectively, is approximately 85.5 degrees, and the distance between the gravity centers of the subunits AB’ and A’B is approximately 58.9 Å. Compared to the concave tetramers in the particles, the structure we obtained is similar to the one that has the smallest deviation and distortion (Figure 3D).

To further investigate the role of the loops, N-, and C-segments in mediating the contacts among the gp105 monomers in the concave tetramers, we made systematic mutagenesis studies on the segments that are involved in mediating the interactions. A total of nine truncation mutants were made, including gp105Δ(NTS 1–17), Δ(NTS 1–60), Δ(CTS 578–631), Δ(NTS 1–17, CTS 609–631), Δ(NTS 1–60, CTS 578–631), Δ(loop 279–285), Δ(NTS 1–60, loop 279–285, CTS 578–631), Δ(NTS 1–60, loop 302–322, CTS 578–631), Δ(NTS 1–60, loop 279–285, loop 302–322, CTS 578–631). SEC analysis of these mutants shows that the truncation mutants on the N- segment 1–60, C- segment 609–631, and loop 279–285, including gp105Δ(NTS 1–17), Δ(NTS 1–17, CTS 609–631), Δ(NTS 1–60), and Δ(loop 279–285) can still form the large aggregates that are eluted from the void volume of the column (Supplementary Figure 6). However, production of the cube-like particles and lattice-like aggregates are significantly reduced by truncations in residues NTS 1–60, loop 279–285, and CTS 609–631, only small peaks of these assemblies were observed in the SEC results (Supplementary Figure 6). These results indicated that residues NTS 1–60, loop 279–285, and CTS 609–631 play a significant but not determinant role in the assembly of gp105. Of note, truncation mutants on the C- segment 578–631, including gp105Δ(NTS 1–60, CTS 578–631), Δ(CTS 578–631), Δ(NTS 1–60, loop 279–285, CTS 578–631), Δ(NTS 1–60, loop 302–322, CTS 578–631), Δ(NTS 1–60, loop 279–285, loop 302–322, CTS 578–631) are all in a monomeric state (Supplementary Figure 6). These data indicated that the C-segment 578–631 that mediates the interactions between molecules A and B is essential for promoting the assembly of gp105.

Our structure is highly similar to the recently published structure of gp105 (chimallin) (PDB ID: 7SQQ) (Laughlin et al., 2022). The published result was focused on analyzing the interactions in the head-to-tail tetramer (Laughlin et al., 2022). However, we are focusing on the concave tetramer of the cube-like assembly and the dynamics of the assembly. Superimpositions of the monomer in the cube-like assembly (PDB ID: 7SQQ) with the two molecules A and B in our concave tetramer showed an R.M.S.D. of 0.75 and 0.82 Å between the aligned Cα atoms, respectively. Differences are observed in several regions, including the N-, and C-terminal segments and loops 273–287, 303–320, 137–155. The C-terminal segment (residues 622–631) is not represented in our map (Supplementary Figure 5B). The loops 273–287, 303–320, and 137–155 all have different conformation in our structure, whereas the loop 303–320 of molecule A has more details with an α helix, which is missing in the recently published structure. In the concave tetramer, loop 273–287 of molecule B also shows different conformation that may be due to its flexibility and ability to adapt to different contacting environments (Supplementary Figure 5B). The structure of gp105 is also similar to the recently reported structures of two homologous proteins, including gp189 of phage Goslar (PDB ID: 7SQT) (Laughlin et al., 2022) and gp53 of phiPA3 (PDB ID: 8FNE) (Nieweglowska et al., 2023). Structural comparisons of gp105 with gp53 and gp189 showed that the differences are mainly in the C- and N-terminal segments and loops (loop 137–155, loop 273–287, loop 303–320) (Supplementary Figure 5C), which are involved in mediating the intermolecular interactions. The comparisons further indicated that the flexibility of the N and C segments and loops 137–155, 273–287, and 303–320 plays a role in adapting to different contacting environments (Supplementary Figure 5C).

### Dynamic assembly of gp105

**FIGURE 6:**
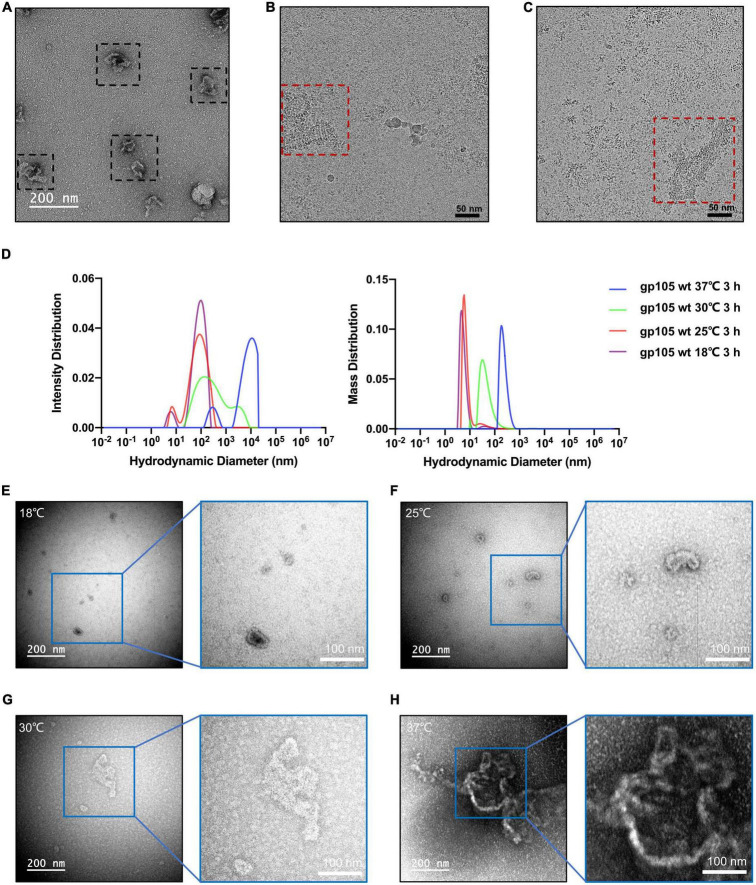
*In vitro* self-assembly of gp105 under different temperatures. **(A)** A representative negative-staining micrograph showing the aggregations generated from gp105 monomers after an incubation of 24 h at 18°C. **(B)** A representative cryo-EM micrograph showing the lattice-like aggregations generated from gp105 monomers after an incubation of 3 h at 18°C. **(C)** A representative cryo-EM micrograph showing the aggregations generated from gp105 monomers after incubation at 30°C for 3 h. **(D)** Dynamic light scattering analysis of the gp105 monomer after incubation for 3 h at different temperatures. The intensity distribution of the gp105 monomer at different temperatures shows the different polymorphic forms in the solution. The mass distribution of the gp105 monomer at different temperatures shows the proportion of different polymorphic forms in the solution. **(E–H)** The representative negative-staining micrographs of gp105 monomer after incubation at 18°C **(E)**, 25°C **(F)**, 30°C **(G)**, and 37°C **(H)** for 3 h.

The SEC and EM analysis showed that the majority of the recombinant gp105s are in a monomeric state. The results are consistent with previous studies on the overexpressed gp105-GFP in cells, which distributes evenly in cells and show no observable aggregations before phage infection (Chaikeeratisak et al., 2017b). In cells, the assembly of gp105 could be controlled by phage-related factors yet to be determined. To investigate possible other factors that may govern the assembling procedure of gp105, we picked the SEC peak fractions of gp105 and investigated the *in vitro* self-assembly of gp105. We found that gp105 monomers can self-assemble to form large aggregates *in vitro* after being incubated at 18°C for several hours and the assemblies have a similar shape and pattern as those eluted from the void volume of the SEC analysis (Figures 6A, B). Induction of the assembly at different temperatures showed that higher temperatures can significantly accelerate the self-assembly of gp105 as shown by the SEC, dynamic light scattering (DLS), and EM analysis of the gp105 samples incubated at different temperatures (Figures 6D–H and Supplementary Figure 7A). However, analysis of the assemblies formed at 30 or 37°C showed no clear lattice-like structures (Figures 6G, H), indicating that the assemblies formed *in vitro* under a high temperature may be more disordered compared to those formed at 18°C (Figures 6B, C).

Size-exclusion chromatography analysis of the collected high-molecular weight gp105 assembly showed that a significant portion of the assemblies can disassemble to form monomeric gp105, indicating that the assembling procedure of gp105 *in vitro* is reversible (Supplementary Figures 7B, C). However, in all these SEC analyses, the peaks containing the cube-like gp105 particles were all small, indicating that the cube-like gp105 particles are not stable and liable to be converted into either the monomeric gp105 or the high molecular weight assembly of gp105 (Supplementary Figures 7B, C), which is consistent with our EM structural analysis.

The high molecular weight assembly of gp105 eluted at the void volume of SEC contains nucleotide contaminations as indicated by the absorption signal at 260 nm. To investigate the possible role of dsDNA in the assembly of gp105, we performed the *in vitro* assembly of gp105 in presence of a dsDNA fragment that encodes gp105. However, the electrophoretic mobility shift assay (EMSA) showed that gp105 does not have specific interactions with dsDNA (Supplementary Figure 1F), dsDNA may not significantly promote the assembly of gp105.

## Discussion

**FIGURE 7:**
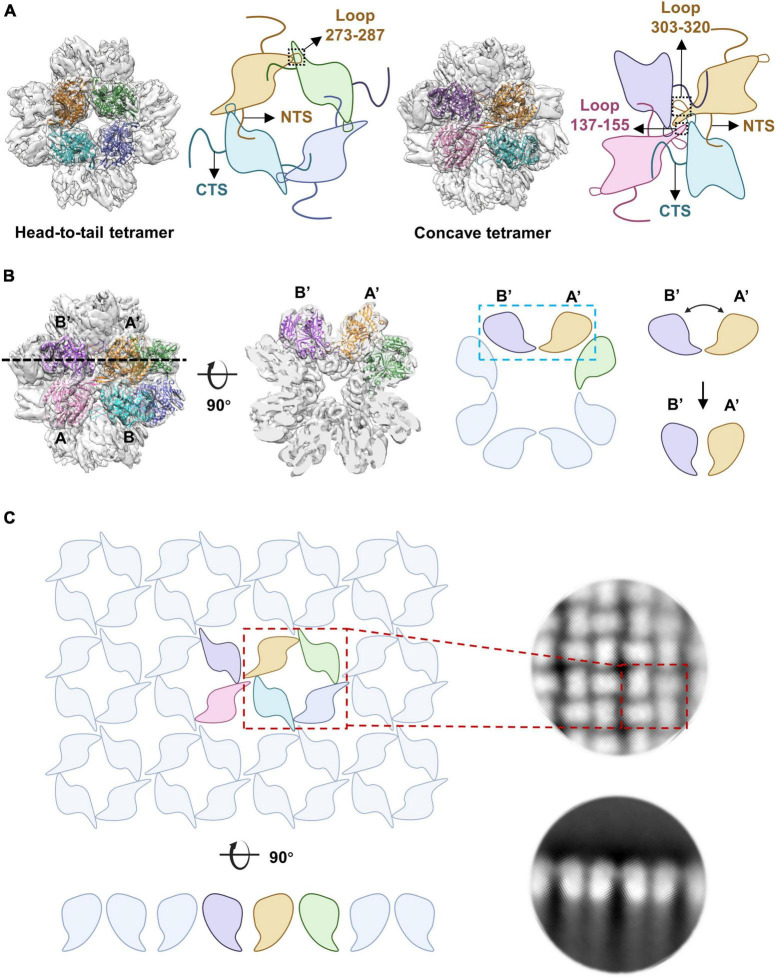
The arrangement of gp105 in the nucleus-like shell. **(A)** Schematic diagrams showing the contacts in the head-to-tail and concave tetramers. (**B**, Left) Surface rendered representations and ribbon diagrams showing the cube-like assembly of gp105 and its cross-section. Each monomer of the fitted head-to-tail and concave tetramers are colored differently. Right: schematic diagrams showing the side view of the concave tetramer and its deformation into the arrangement required for the formation of the lattice-like structure, through the change of the bending angle between AB’ and A’B. **(C)** Schematic diagrams showing the organization of gp105 in the lattice-like structure. Right is the 2D class averages of the highly aggregated gp105 eluted at the void volume of SEC.

Our structural and biochemistry data showed that the assembly of the nucleus-like shell (NLS) mainly depends on the formation of the head-to-tail and concave tetramers of gp105 (Figure 7A). In the tetramers, the NTS and CTS of gp105 play an essential role in mediating the interactions between the monomers. In the cube-like assembly of gp105, the middle ring of the particle is relatively stable, but the tetramers at the distal end are liable to disassemble to form the monomers or alter the bending angles between the subunits of the concave tetramers, which helps to change the curvature of the assembly and facilitate the incorporation of additional subunits to form larger particles or the lattice-like assemblies (Supplementary Figure 7D). The cube-like particles are likely the intermediate state in the assembly of gp105.

The 2D averaged images showed particles that have an elongated shape with a long axis of 320 Å, which is approximately 1.5 times of the length of the cube-like particles. These particles appear to be the merged form of two cube-like particles (Supplementary Figure 7E) as have been described (Laughlin et al., 2022). By changing the bending angle between the subunits AB’ and A’B of the concave tetramer, the curvature of the assembly can be regulated. When the bending angle is approximately 90 degrees, gp105 forms cube-like particles, which is the smallest form of the nucleus-like shell (Figure 7B). When the subunits AB’ and A’B of the concave tetramers are almost parallel to each other, a lattice-like plane of the nucleus-like shell can be formed (Figures 7B, C). Thus, the concave tetramers play a key role in regulating the size and curvature of the gp105 assemblies.

The assembly of gp105 *in vitro* at 18°C can form well-ordered lattice-like structures that presumably have the same architecture as in the nucleus-like shell (Laughlin et al., 2022). Although higher temperatures can significantly increase the assembly rate of gp105 *in vitro*, clearly lattice-like features were barely observed in the assemblies formed at 30 or 37°C. It was shown that the overexpressed gp105 did not self-assemble in the cell prior to phage infection although the cell culture temperature is 30°C, under which the gp105 *in vitro* can quickly form large aggregates. Thus, initiation of the assembly of gp105 in cells must involve other phage-encoded factors. In the meantime, the crowded environment or other host or phage-encoded factors may be involved in directing the precise assembly of gp105 in cells, so that the well-ordered nucleus-like shell can be assembled under the culture temperature of 30°C. In addition, these factors may also function in preventing the disassembly of gp105, which can occur *in vitro* but not in cells.

There are two forms of pores on the lattice-like assemblies, including the center pore and the auxiliary pore (Supplementary Figure 8A). Although the size of the pores in different class-averaged images varies, the average size of the center pore is less than 22 Å, while the auxiliary pore is larger and has an average size of approximately 30 Å (Supplementary Figures 8A, B). The pores are big enough for the passage of mRNA or dsDNA but may not big enough for the translocation of globular proteins that have a molecular weight of more than 10 kDa (Erickson, 2009). However, it was reported that the nucleus-like structure of phage ΦKZ can selectively import molecules that normally reside in the cytoplasm with a molecular weight of more than 100 kDa by fusing a mutant of GFP (Nguyen et al., 2021). It was proposed that the genome dsDNA of jumbo phages is synthesized in the nucleus, transported out of the nucleus-like structure, and then packaged into the procapsid shells that are attached to the nucleus-like shell (Chaikeeratisak et al., 2017b,^2019^). The transportation of the dsDNA phage genome depends on an ATP-dependent complex machinery, of which an essential component is the portal dodecamer (Fokine and Rossmann, 2014). Assembly of the DNA packaging machinery on the nucleus-like shell and transportation of proteins with a large molecular weight through the nucleus-like shell may depend on the larger pores that are formed by gp105 and other components of the nucleus-like shell, which needs further studies.

## Materials and methods

### Gene synthesis and cloning

The gene encoding the major nuclear shell protein gp105 of the Pseudomonas phage 201phi2-1, (GenBank: ABY62937) was synthesized (Qinglan Biotech, Wuxi, China) with codon optimization for expression in *E. coli* cells. The synthesized gene was cloned into the pET28a vector with a 6 × His tag in fusion with the C-terminus of gp105. The plasmid was transformed into *E. coli* DH5α for amplification.

The truncation mutants were generated through PCR and In-Fusion cloning by using a one-step cloning kit (Vazyme, #C112-01) and were also inserted into the pET28a vector.

### Protein expression and purification

The plasmids were transformed into *E. coli* BL21 (DE3) pLysS cells (Thermo Fisher Scientific, Waltham, MA, USA) for producing the recombinant proteins. Protein expression was induced by adding 1 mM isopropyl β-D-1-thiogalactopyranoside (IPTG) at 16°C. Cells were harvested by centrifugation at 4,000 × *g* for 15 min. The pellet was collected and resuspended in buffer A (20 mM HEPES at pH 7.5, 300 mM NaCl) with 1 mM PMSF. The resuspended cells were lysed through ultrasonication and cell debris was pelleted by centrifugation at 12,000 × *g* for 30 min. The supernatant containing the recombinant protein was applied to cobalt resins (Takara, #635653) for affinity purification. The elution from the cobalt resins was collected, concentrated and then further purified with a Superose 6 Increase 10/300 GL column (GE Healthcare, #29-0915-96) running in buffer B (20 mM HEPES at pH 7.5, 150 mM NaCl). SDS-PAGE gel analysis showed that all three elution peaks from the size-exclusion chromatography carry only gp105. All the truncation mutants were expressed and purified using the same method as for the wild type gp105. For analysis by size exclusion chromatography coupled with multi-angle light scattering (SEC-MALS) (Wyatt Technology), a 100 μl sample of wild type gp105 at 2 mg/ml was passed over a Superose 6 Increase 10/300 GL column (GE Healthcare, #29-0915-96) in buffer B. The molecular weight was calculated by using ASTRA v7.3.2 software (Wyatt Technology).

### Negative-staining electron microscopy

For negative-staining sample preparation, 5 μl of the sample at a concentration of 0.02–0.1 mg/ml was applied onto a glow-discharged grid with a continuous carbon layer. The samples were incubated on the carbon grid for 1 min, the excess sample was removed with filter paper and washed twice with 5 μl of 2% uranyl acetate (UA) and then incubated with 5 μl of 2% UA for 45 s. The excess UA was removed with filter paper and then the grids were air-dried at room temperature. All the grids prepared were examined with a 120 kV FEI Tecnai Spirit electron microscope.

### Cryo-electron microscopy sample preparation and data collection

3.5 μl peak elution from the size-exclusion chromatography was applied to glow-discharged holey carbon grids (Quantifoil, Cu 200 mesh, R1.2/1.3) at a concentration of 0.5-0.8 mg/ml. The grids were blotted for 4.5 s in 100% humidity at 8°C and plunged into liquid ethane immediately by using Vitrobot Mark VI (Thermo Fisher).

The cryo-EM data of gp105 cube-like particles and gp105 aggregation eluted from the void volume of the SEC were collected on a 300 kV Titan Krios that is equipped with a Gatan K3 Summit camera. The defocus values were set in the range of –1.2 μm to –2.8 μm and the total dose used was ∼50 electrons per Å^2^. A total of 6,048 movie stacks were collected for the cube-like particles at a magnification of 22,500, which yield a calibrated pixel size of 0.625 Å (Supplementary Table 1). A total of 2,930 movie stacks were collected for the gp105 aggregation eluted from the void volume of the SEC at a magnification of 29,000, which yield a calibrated pixel size of 0.485 Å (Supplementary Table 1).

The cryo-EM data of gp105 aggregates formed by incubating gp105 monomer under different temperatures were collected on a 200 kV FEI Talos Arctica that is equipped with a Gatan K2 Summit camera. The data of the gp105 monomer incubated at 18°C for 3 h were collected with a magnification of 36,000, which yield a calibrated pixel size of 0.585 Å. The data of the gp105 monomer incubated at 30°C for 3 h were collected with a magnification of 45,000, which yield a calibrated pixel size of 0.47 Å.

### Image processing and structure refinement

Images of each stack were aligned, summed, and 2 × binned using the program Motion Cor2 (Zheng et al., 2017). Micrograph CTF parameters were determined using the Gctf program (Zhang, 2016) with local variations of the defocus taken into consideration. Further processing of the data was performed with the program RELION-v3.1.0 and RELION-v3.0.8 (Scheres, 2012). The image processing procedures are summarized in Supplementary Figure 4.

For the dataset of the cube-like particles, particles were boxed by using RELION-v3.1.0 Autopick and Gautomatch-v0.56.^1^ At the initial stage of the calculation, a small number of particles (80,754 particles) were selected by using RELION-v3.1.0 Autopick. These particles were used for generating the initial model with C1 symmetry by using the 3D initial model program in RELION. The model was further improved by 3D auto-refinements with ether Dihedral (D), octahedral (O), or tetrahedral (T) symmetry imposed. Only the O symmetry gave a reasonable result. Then, 654,844 particles were boxed by using Gautomatch-v0.56 with the averaged images from the initial 80,754 particles as references. After several rounds of 2D classifications, particles in classes that did not have detailed features were removed. After the 2D classifications, 291,748 particles were selected from 6,048 images. The selected 291,748 particles were then subjected to multiple-reference 3D classifications with C1 symmetry. After the 3D classifications, 134,458 particles were selected for further 3D auto-refinement with or without O symmetry imposed. The reconstruction with O symmetry imposed yielded a density map at a resolution of 4.76 Å. The reconstruction without applying any symmetry yielded a density map at a resolution of 6.41 Å. The refined half maps were further processed with deepEMhancer (Sanchez-Garcia et al., 2021) or RELION.

To further improve resolution, we used focused reconstruction by using the block-based algorithm (Zhu et al., 2018) with the orientations and centers from the O symmetry imposed reconstruction. The concave tetramer blocks on the 2-fold axis were created with a local mask generated from the fitted atomic models. The spatial coordinates of the center of the concave tetramer block were determined by using UCSF Chimera and EMAN1 (Ludtke et al., 1999). The block-based reconstruction script BBR_with_relion_v9.py was used to determine the spatial coordinates of the equivalent blocks (Zhu et al., 2018). The script split_star_for_preprocessing.py was used to generate the blocks based on the different equivalent points. The particle extraction function of RELION-v3.0.8 was used to re-extract the particles with a box size of 160 Å. RELION reconstruction was used to generate the initial model of the block. The sub-particles of the blocks were subjected to 3D classifications with C2 symmetry imposed and 290,996 particles were selected for the final 3D auto-refinement, which resulted in a map with a resolution of 4.09 Å. Prior to visualization, all density maps were sharpened by using the RELION post-processing program and the maps (O map, C1 map) for analysis were further processed by using deepEMhancer (Sanchez-Garcia et al., 2021). The reported resolutions are all based on the gold-standard Fourier shell correlation (FSC) with the criterion set at 0.143 (Rosenthal and Henderson, 2003).

For the dataset of the gp105 aggregation eluted from the void volume of the SEC, particles were boxed from 2,930 images using the AutoPick from RELION-v3.1.0, After several rounds of 2D classifications, 164,497 particles (4 × binned) with a box size of 320 Å were selected. For analyzing molecules at the edge of the aggregations, 55,669 particles were boxed and selected for 2D classifications. The image processing procedures are summarized in Supplementary Figure 2.

### Model building

We used AlphaFold (Jumper et al., 2021) to generate a reference model of the nucleus-like shell protein (NLSP) gp105, which was then used for model building. The predicted gp105 model was fitted into the concave tetramer map using UCSF Chimera (Pettersen et al., 2004) and then the fitted model was manually adjusted in COOT (Emsley et al., 2010). Real-space refinements of the adjusted model were performed with PHENIX (Adams et al., 2010). The models were improved by several rounds of manual adjustments and refinements. Residues 1–48 at the N terminus, residues 274–286, 315–319 in the loop regions, residues 583–631 at the C terminus of molecule A and residues 1–62 at the N terminus, residues 138–154, 304–319 in the loop regions, residues 585–588, 611–631 at the C terminus of molecule B were not built due to the highly disordered densities. The refinement statistics for the models were summarized in Supplementary Table 1.

The correlation coefficients (CCs) between the maps were calculated using UCSF Chimera (Pettersen et al., 2004), After fitting the map with C1 symmetry into the map with O symmetry by maximizing the overall CC, the correlation coefficient was then calculated separately for each molecule and the map in Figure 3 was colored according to the numerical magnitude of the CC values. All numerical calculations of the angles and distances were performed by using UCSF Chimera. The planes or axes of the models were generated with the Structure Measurements function of the UCSF Chimera. All figures were generated with UCSF Chimera.

### Dynamic light scattering analysis

Dynamic light scattering (DLS) analysis of the samples at different conditions was performed with the instrument Uncle (Unchained Labs, Norton, MA, USA). The monomeric gp105 was incubated for 3 h at 18, 25, 30, and 37°C, respectively and the samples were then analyzed with the instrument Uncle (Unchained Labs, Norton, MA, USA). Samples were centrifuged at 12,000 × *g* for 10 min and mixed well before being loaded into disposable cuvettes. Dynamic light scattering data were analyzed with the Uncle Analysis (Unchained Labs, Norton, MA, USA).

### Electrophoretic mobility shift assay (EMSA)

The dsDNA fragment (100 ng) that encodes gp105 was incubated with different amounts of gp105 (0, 10, 50, 100, 200, 500, 1000 ng) at 30°C for 30 min. The samples were then analyzed by agarose gel electrophoresis (1% agarose gel, 120 V, 20 min).

## Data availability statement

The datasets presented in this study can be found in online repositories. The names of the repository/repositories and accession number(s) can be found below: http://www.wwpdb.org/, 8IGG; https://www.ebi.ac.uk/pdbe/emdb/, EMD-35430–EMD-35432.

## Author contributions

ZL planned and performed the experiments, analyzed the data, prepared the figures, and wrote the manuscript. YX planned and supervised the experiments, analyzed the data and wrote the manuscript. Both authors contributed to the article and approved the submitted version.

## Footnotes

1
https://www2.mrc-lmb.cam.ac.uk/download/gautomatch-056/
