# Supplementary material for: Structural studies of the nucleus-like assembly of jumbo bacteriophage 201φ2-1
Source: Front Microbiol. 2023 Apr 17;14:1170112. doi: 10.3389/fmicb.2023.1170112 (PMC10149743; doi:10.3389/fmicb.2023.1170112)
Supplement: Supplementary file 1 [file Data_Sheet_1.pdf]

Supplementary Material

Structural studies of the nucleus-like assembly of jumbo bacteriophage 201φ2-1

Zhe Liu, Ye Xiang*

*** Correspondence:**
To whom correspondence should be addressed: Y.X.: yxiang@mail.tsinghua.edu.cn, Tel: +86-10-62772587

1 Supplementary Figures and Table

1.1 Supplementary Figures

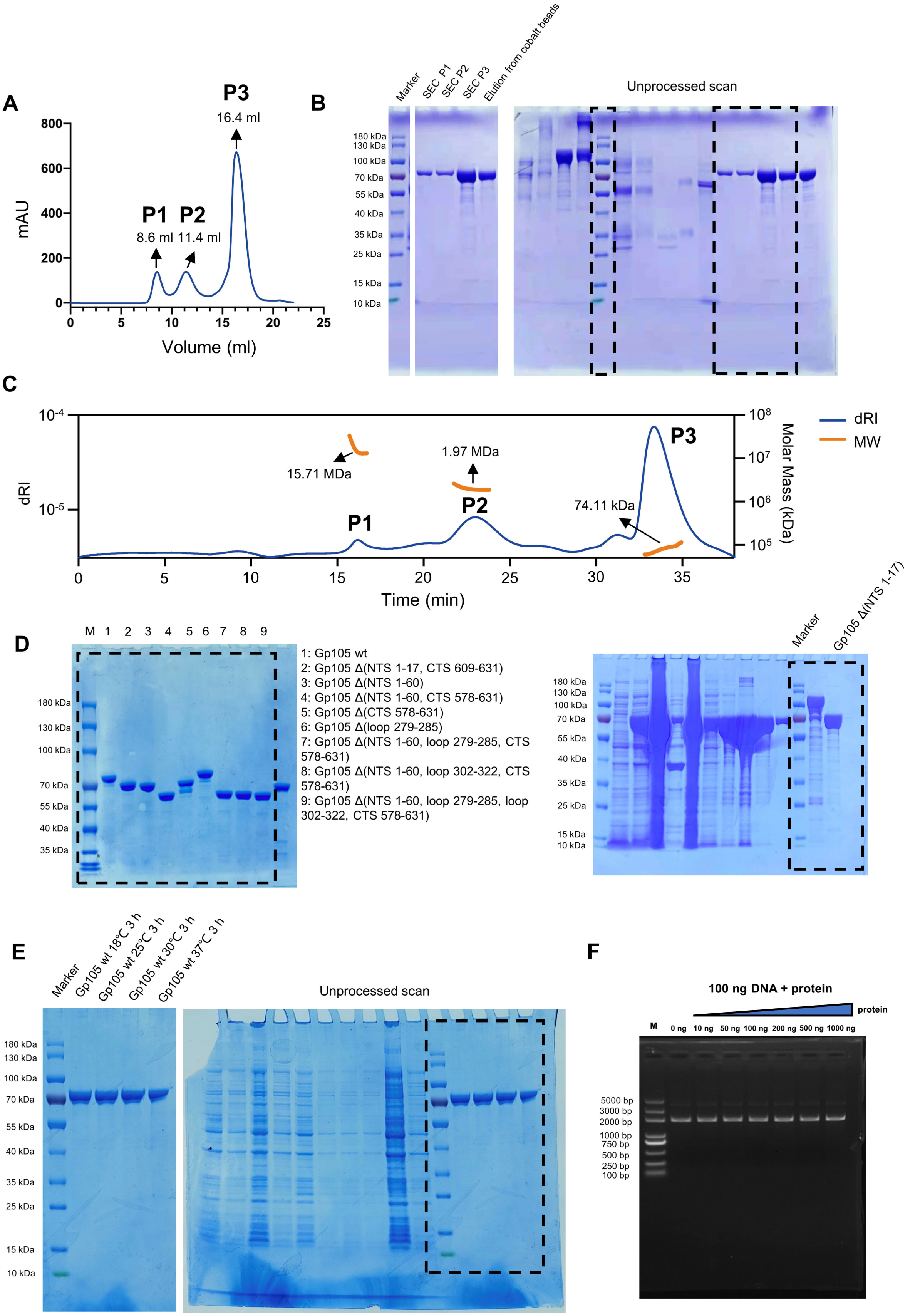
A
P3
16.4 ml
P1 P2
8.6 ml 11.4 ml
mAU
800
600
400
200
0
0 5 10 15 20 25
Volume (ml)
B
Marker
SEC P1
SEC P2
SEC P3
Elution from cobalt beads
Unprocessed scan
180 kDa
130 kDa
100 kDa
70 kDa
55 kDa
40 kDa
35 kDa
25 kDa
15 kDa
10 kDa
C
10^{-4}
10^{-5}
dRI
15.71 MDa
1.97 MDa
74.11 kDa
P1
P2
P3
10^{8}
10^{7}
10^{6}
10^{5}
Molar Mass (kDa)
dRI
MW
0 5 10 15 20 25 30 35
Time (min)
D
M 1 2 3 4 5 6 7 8 9
1: Gp105 wt
2: Gp105 Δ(NTS 1-17, CTS 609-631)
3: Gp105 Δ(NTS 1-60)
4: Gp105 Δ(NTS 1-60, CTS 578-631)
5: Gp105 Δ(CTS 578-631)
6: Gp105 Δ(loop 279-285)
7: Gp105 Δ(NTS 1-60, loop 279-285, CTS 578-631)
8: Gp105 Δ(NTS 1-60, loop 302-322, CTS 578-631)
9: Gp105 Δ(NTS 1-60, loop 279-285, loop 302-322, CTS 578-631)
Marker
Gp105 Δ(NTS 1-17)
E
Marker
Gp105 wt 18°C 3 h
Gp105 wt 25°C 3 h
Gp105 wt 30°C 3 h
Gp105 wt 37°C 3 h
Unprocessed scan
F
100 ng DNA + protein
protein
M
0 ng 10 ng 50 ng 100 ng 200 ng 500 ng 1000 ng
5000 bp
3000 bp
2000 bp
1000 bp
750 bp
500 bp
250 bp
100 bp

Supplementary Figure 1. Purification and biochemistry characterization of gp105. (A) Size exclusion chromatography (SEC) elution profile of the full-length gp105. (B) SDS-PAGE analysis of the full-length gp105 purified by cobalt resins and SEC. (C) Size exclusion coupled with multi-angle light scattering (SEC-MALS) analysis of the purified gp105. From left to right, the measured molar mass of the three peaks are 15.71±0.06 MDa, 1.97±0.01 MDa, and 74.11±1.69 kDa, respectively. (D) SDS-PAGE gel analysis of the purified gp105 truncations. (E) SDS-PAGE gel analysis of the full-length gp105 after being incubated at different temperatures. The full-length gp105 protein was incubated at 18, 25, 30, and 37°C for 3 hours, respectively. (F) Electrophoretic mobility shift assay (EMSA) of dsDNA with full-length gp105. The dsDNA fragment that encodes gp105 (100 ng) was incubated with different amounts (0, 10, 50, 100, 200, 500, 1000 ng) of full-length gp105 at 30°C for 30 minutes.

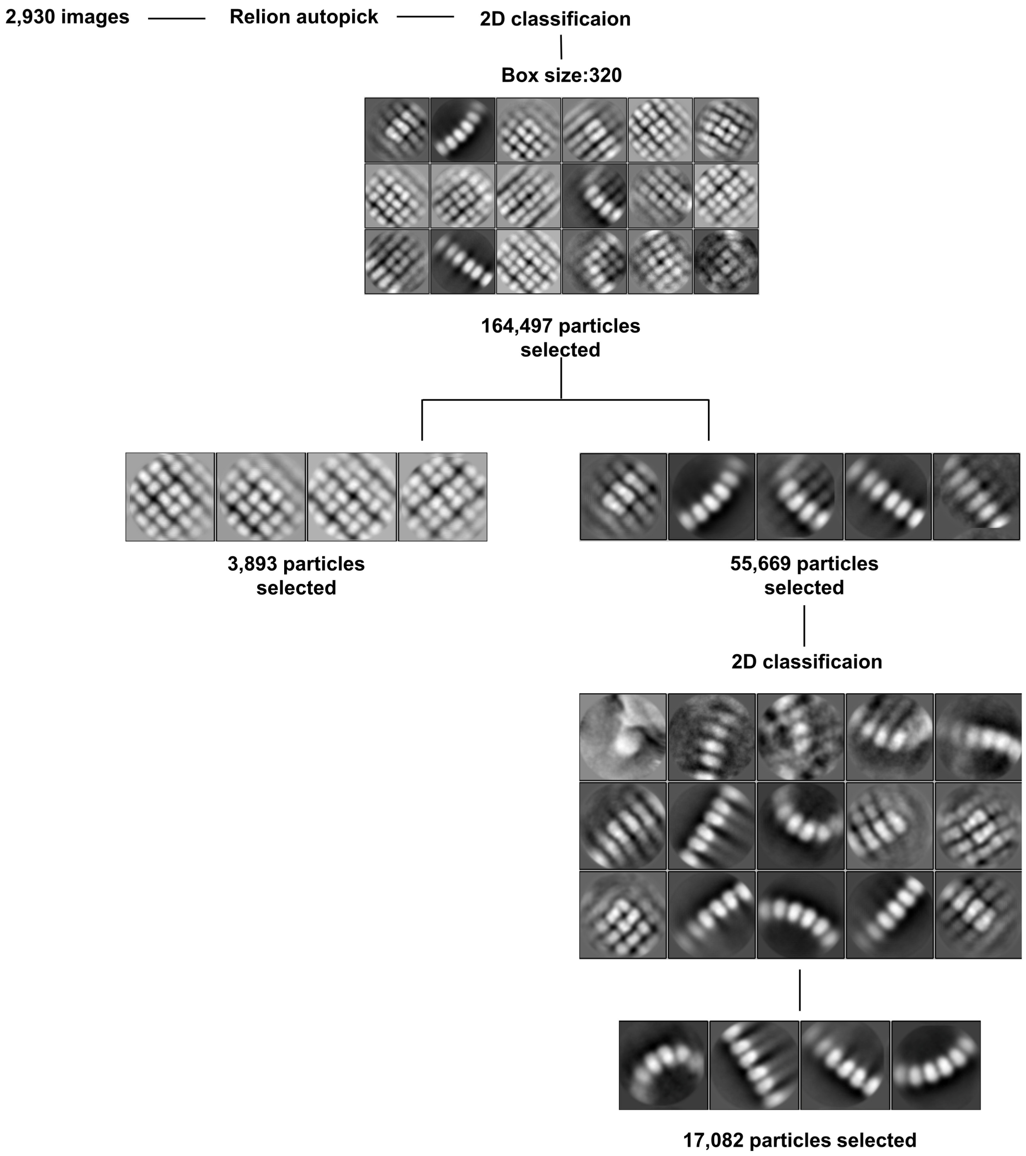

Supplementary Figure 2. Cryo-EM data processing workflow of the highly aggregated gp105 eluted at the void volume of SEC. Particles pick, extraction, and 2D classification were performed using RELION-v3.1.0.

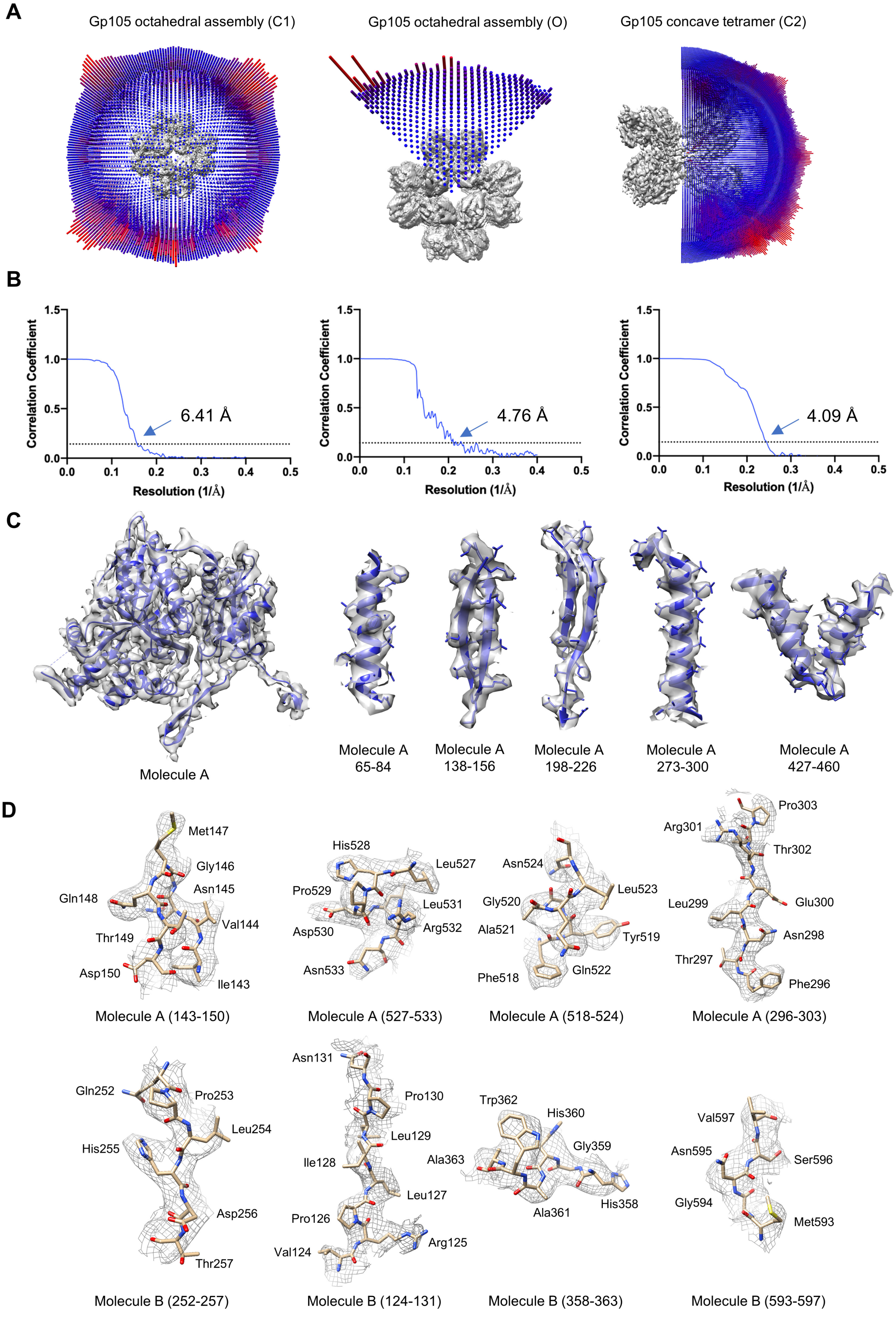
A
Gp105 octahedral assembly (C1)
Gp105 octahedral assembly (O)
Gp105 concave tetramer (C2)
B
Correlation Coefficient
Resolution (1/Å)
6.41 Å
4.76 Å
4.09 Å
C
Molecule A
Molecule A 65-84
Molecule A 138-156
Molecule A 198-226
Molecule A 273-300
Molecule A 427-460
D
Met147
Gly146
Asn145
Gln148
Val144
Thr149
Asp150
Ile143
Molecule A (143-150)
His528
Leu527
Pro529
Leu531
Asp530
Arg532
Asn533
Molecule A (527-533)
Asn524
Leu523
Gly520
Ala521
Tyr519
Phe518
Gln522
Molecule A (518-524)
Pro303
Arg301
Thr302
Leu299
Glu300
Asn298
Thr297
Phe296
Molecule A (296-303)
Gln252
Pro253
Leu254
His255
Asp256
Thr257
Molecule B (252-257)
Asn131
Pro130
Leu129
Ile128
Leu127
Pro126
Arg125
Val124
Molecule B (124-131)
Trp362
His360
Gly359
Ala363
Ala361
His358
Molecule B (358-363)
Val597
Asn595
Ser596
Gly594
Met593
Molecule B (593-597)

Supplementary Figure 3. Cryo-EM data processing and reconstruction results of gp105. (A) Orientation distribution of the particles used for the final refinements of the gp105 cube-like and concave tetramer assemblies. (B) Fourier shell coefficient (FSC) curves of the reconstructions. The FSC threshold is 0.143 and the xml files are from the RELION post-processing results. (C) The molecule A of the gp105 concave tetramer in the density map. The density map is contoured at 4.3 σ. The model is shown in a ribbon diagram. (D) Representative densities of the gp105 concave tetramer assembly. All the density maps are shown as grep mesh. The maps are contoured at 4.3 σ. The models are shown in sticks with C, O, and N atoms colored yellow, red, and blue, respectively.

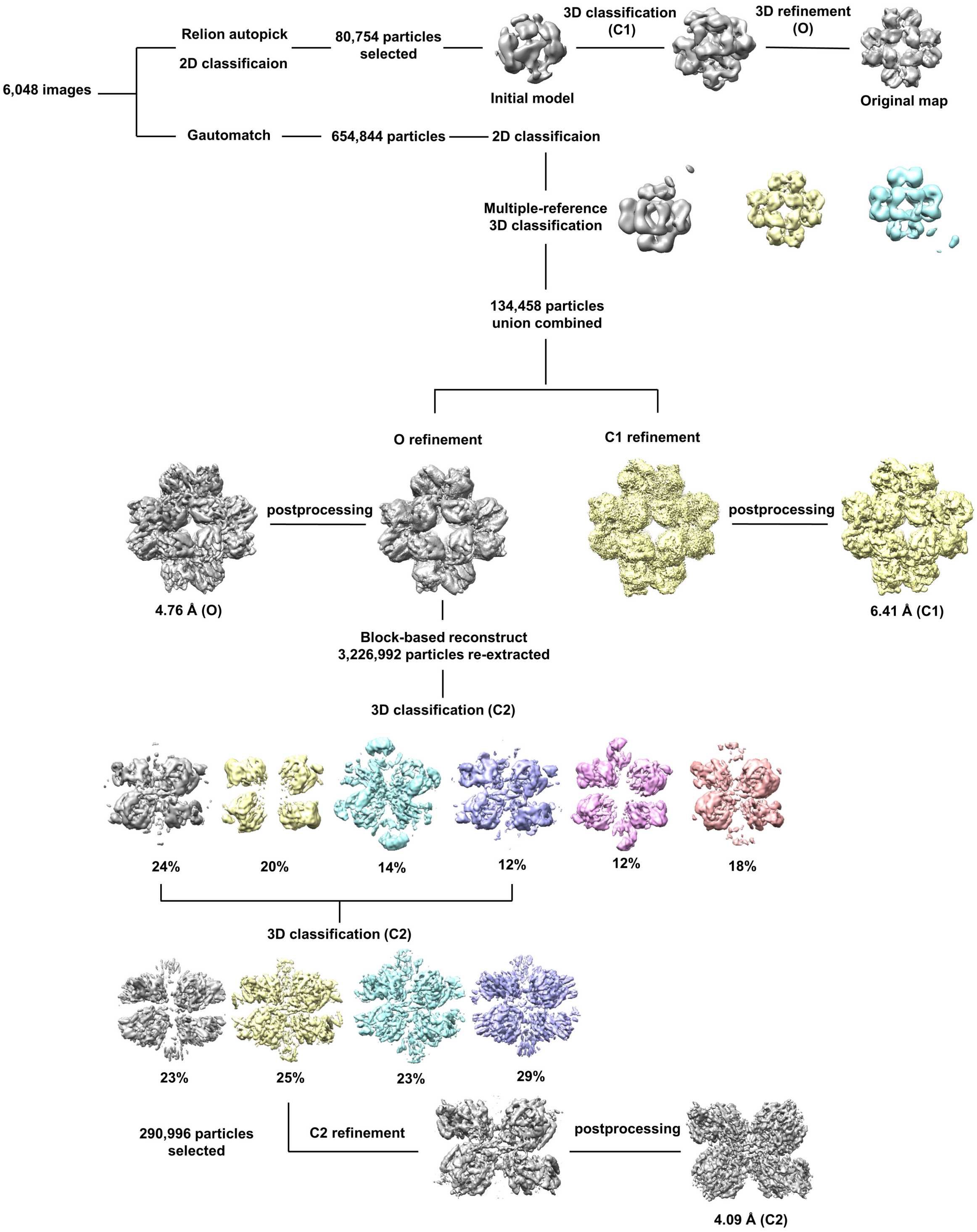

Supplementary Figure 4. The flowchart of the gp105 data processing procedure. Particles were picked by RELION-v3.1.0 Autopick and Gautomatch-v0.56. Particle extraction, 2D classification, 3D classification, and 3D refinement were performed using RELION-v3.1.0. The focused refinements on the concave tetramer were performed using RELION-v3.0.8. The refined half maps were subjected to the RELION post-processing and deepEMhancer for post-processing.

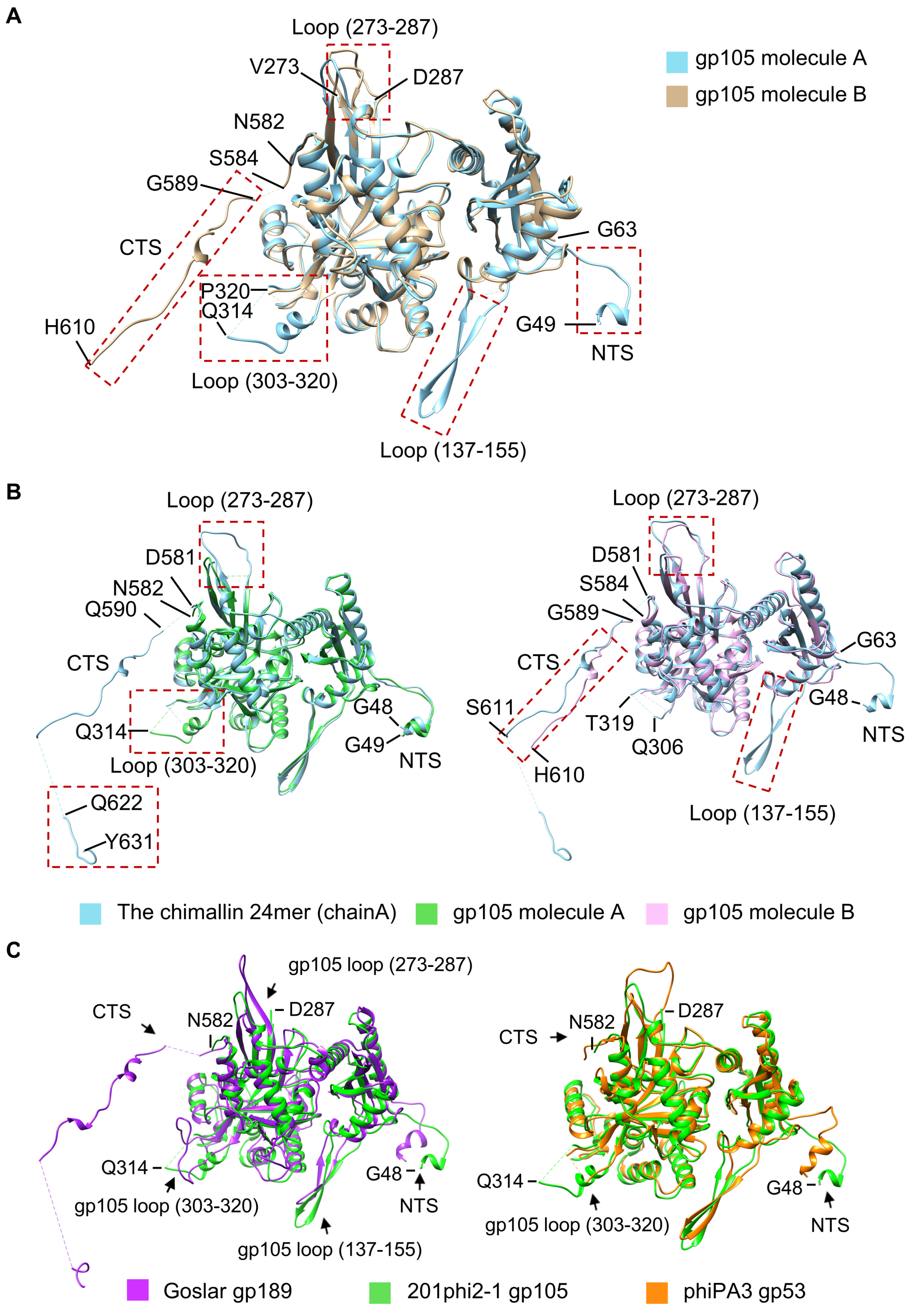

Supplementary Figure 5. (A) Structural comparisons of the gp105 molecules A and B in the concave tetramer. (B) Structural comparisons of the chimallin 24mer chain A (PDB ID: 7SQQ) and the gp105 molecules A and B. (C) Structural comparisons of the gp105 molecule A and the homologous structures, including the gp189 of phage Goslar (PDB ID: 7SQT) and the gp53 of phage phiPA3 (PDB ID: 8FNE). The black arrows show the sites of possible interactions between molecules.

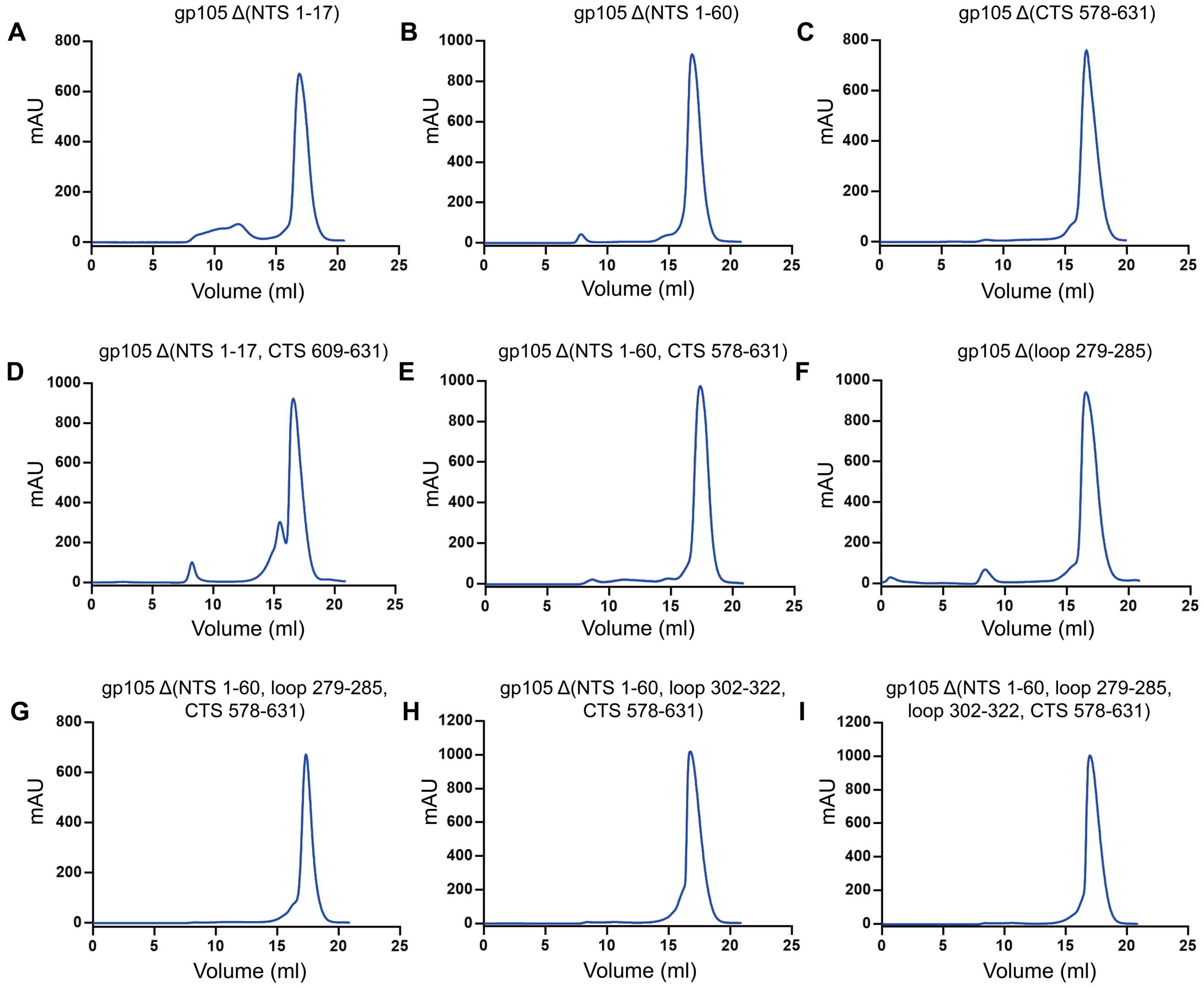

Supplementary Figure 6. Size exclusion chromatography (SEC) elution profiles of different gp105 truncations. SEC analysis of (A) gp105Δ(NTS 1-17), (B) gp105Δ(NTS 1-60), (C) gp105Δ(CTS 578-631), (D) gp105Δ(NTS 1-17, CTS 609-631), (E) gp105Δ(NTS 1-60, CTS 578-631), (F) gp105Δ(loop 279-285), (G) gp105Δ(NTS 1-60, loop 279-285, CTS 578-631), (H) gp105Δ(NTS 1-60, loop 302-322, CTS 578-631), (I) gp105Δ(NTS 1-60, loop 279-285, loop 302-322, CTS 578-631).

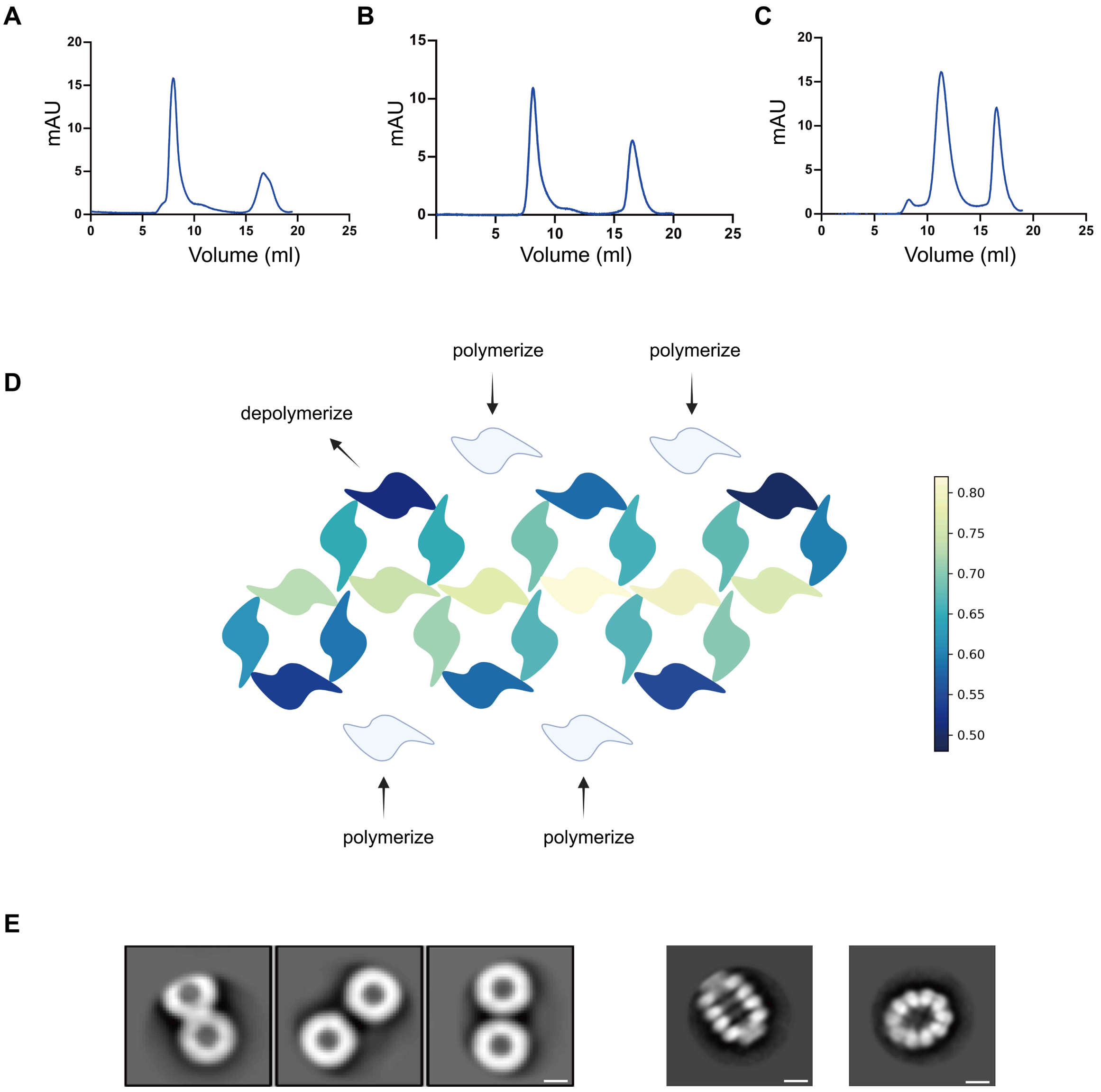

Supplementary Figure 7. Dynamic assembly of gp105. Size exclusion chromatography (SEC) re-analysis of different SEC peaks of the full-length gp105 treated under different conditions. (A) The peak 3, which is in the monomeric state, was incubated at 37°C for 1 h and then analyzed with SEC again. (B) The peak 1 from SEC, which is highly aggregated gp105 eluted at the void volume, was incubated at 4°C for 24 h and then analyzed with SEC again. (C) The peak 2, which contains the cube-like particles, was incubated at 4°C for 24 h and then analyzed with SEC again. (D) Schematic diagram of the dynamic assembly of gp105. The free molecules in solution are colored light blue and the middle is the floor plane of the gp105 cube-like assembly. The dark blue molecules may depolymerize or recruit other free molecules to assemble. (E) 2D class averages of the gp105 cube-like assembly showing the particles formed by two merged cube-like particles. The scale bars represent 10 nm.

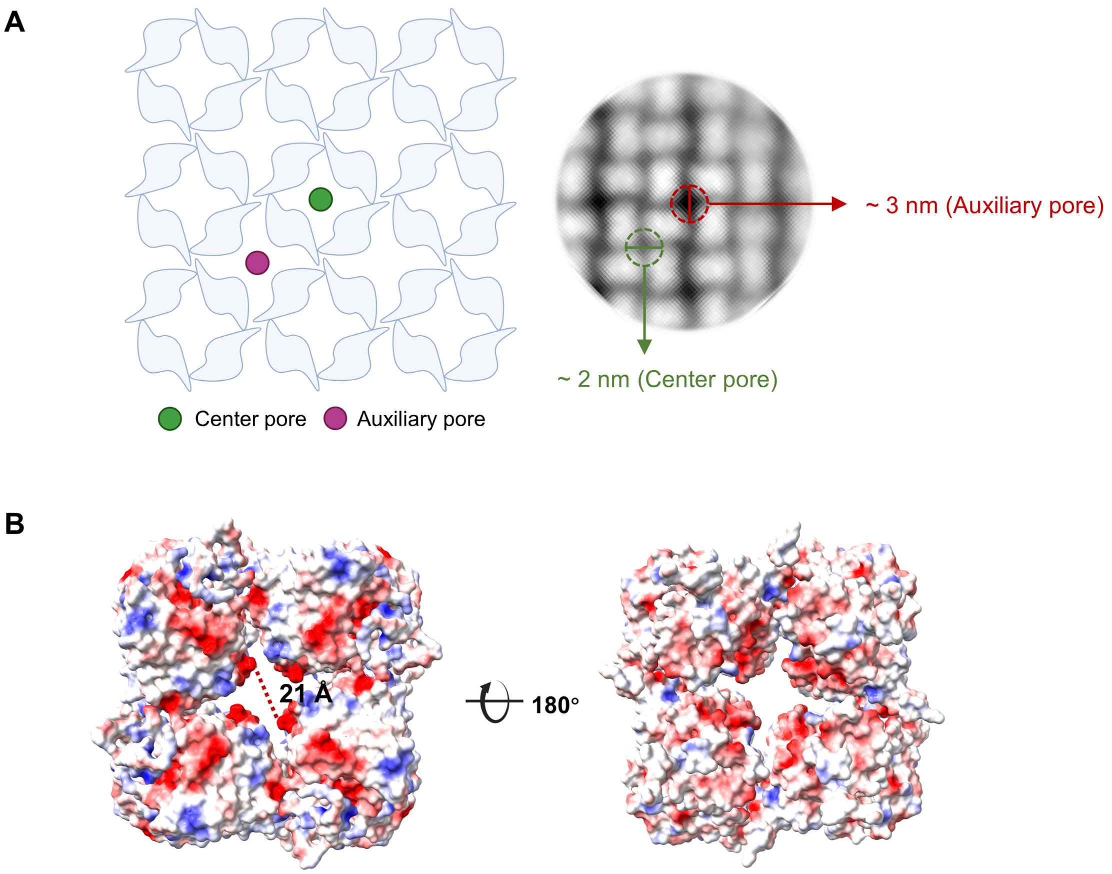

Supplementary Figure 8. The pores of the nucleus-like shell. (A) Schematic diagram of the lattice-like structure showing two types of holes in the lattice, including the center pore (green sphere) and the auxiliary pore (dark pink sphere). (B) Surface rendered diagrams showing the surface electrostatic potential of the head-to-tail tetramer (The fitted gp105 molecule A in the cryo-EM map of the gp105 O map). The surface is colored according to the surface electrostatic potential with red representing negative electrostatic potential and blue representing positive electrostatic potential. The size of the center pore is approximately 21 Å.

1.2 Supplementary Table

Supplementary Table 1. Cryo-EM data collection and image processing statistics.

Sample	SEC peak 2			SEC peak 1
	concave tetramer	cube-like particle (O)	cube-like particle (C1)	sheet-like assembly
Acceleration voltage (kV)	300			
Detector	K3 Summit			
Electron exposure ($e^-/Å^2$)	50			
Magnification	22,500			29,000
Pixel size (Å)	1.25			0.97
Symmetry imposed	C2	O	C1	
Particles used in the final refinement	290,996	134,458		
Map resolution (Å)	4.09	4.76	6.41	
Map sharpening B factor ($Å^2$)	-206.429	-115.372	-237.111	
EMDB ID	EMD-35432	EMD-35431	EMD-35430	
PDB ID	8IGG			
R.m.s. deviations (bond lengths) (Å)	0.004			
R.m.s. deviations (bond angles) (°)	0.991			
Molprobity score	1.98			
Correlation coefficient (CCmask)	0.82			
Rotamer outliers (%)	0			
CaBLAM outliers (%)	4.70			
Clashscore	9.75			
Ramachandran favored (%)	92.60			
Ramachandran allowed (%)	7.40			
Ramachandran outliers (%)	0			
